# Modeling Snyder-Robinson Syndrome in multipotent stromal cells reveals impaired mitochondrial function as a potential cause for deficient osteogenesis

**DOI:** 10.1038/s41598-019-51868-5

**Published:** 2019-10-28

**Authors:** Ashley L. Ramsay, Vivian Alonso-Garcia, Cutter Chaboya, Brian Radut, Bryan Le, Jose Florez, Cameron Schumacher, Fernando A. Fierro

**Affiliations:** 1 Institute for Regenerative Cures, University of California Davis, 2921 Stockton Blvd, Sacramento, CA USA; 2 Department of Cell Biology and Human Anatomy, University of California, Davis, USA

**Keywords:** Musculoskeletal models, Mesenchymal stem cells

## Abstract

Patients with Snyder-Robinson Syndrome (SRS) exhibit deficient Spermine Synthase (SMS) gene expression, which causes neurodevelopmental defects and osteoporosis, often leading to extremely fragile bones. To determine the underlying mechanism for impaired bone formation, we modelled the disease by silencing SMS in human bone marrow - derived multipotent stromal cells (MSCs) derived from healthy donors. We found that silencing SMS in MSCs led to reduced cell proliferation and deficient bone formation *in vitro*, as evidenced by reduced mineralization and decreased bone sialoprotein expression. Furthermore, transplantation of MSCs in osteoconductive scaffolds into immune deficient mice shows that silencing SMS also reduces ectopic bone formation *in vivo*. Tag-Seq Gene Expression Profiling shows that deficient SMS expression causes strong transcriptome changes, especially in genes related to cell proliferation and metabolic functions. Similarly, metabolome analysis by mass spectrometry, shows that silencing SMS strongly impacts glucose metabolism. This was consistent with observations using electron microscopy, where SMS deficient MSCs show high levels of mitochondrial fusion. In line with these findings, SMS deficiency causes a reduction in glucose consumption and increase in lactate secretion. Our data also suggests that SMS deficiency affects iron metabolism in the cells, which we hypothesize is linked to deficient mitochondrial function. Altogether, our studies suggest that SMS deficiency causes strong transcriptomic and metabolic changes in MSCs, which are likely associated with the observed impaired osteogenesis both *in vitro* and *in vivo*.

## Introduction

Snyder-Robinson Syndrome (SRS) is an X-linked recessive condition characterized by mental retardation, skeletal defects, hypotonia, and movement disorders^1^. In all reported cases, SRS has been associated with mutations leading to dysfunction of Spermine synthase (SMS), which catalyzes the conversion of spermidine into spermine and is therefore an important enzyme of the polyamine pathway^2^. Recently, Li *et al*. modelled SRS in Drosophila and found that SMS deficiency causes lysosomal dysfunction and oxidative stress^3^.

To date the causes for debilitated bones in SRS patients are unknown. They may include dysregulation of parathyroid hormone, calcium metabolism, or other systemic effects. Alternatively, weak bones could be caused by osteoclast hyperactivity or dysfunction of osteo-progenitor cells (MSCs)^4^. Albert *et al*. evaluated these parameters in two SRS patients and found that low bone density is likely the consequence of a failure to mineralize due to poor differentiation of MSCs into osteoblasts^5^. However, the low number of individuals tested, and intrinsic donor-to-donor variations made more general conclusions challenging. Most important, the underlying molecular consequences that could explain the defects in osteogenesis due to deficient SMS activity have not been investigated.

Studying MSCs derived from SRS patients is challenged by multiple factors. First, the number of identified patients is very low. Second, the procedure to obtain bone marrow is very invasive, especially considering the fragility of the patient’s bones. Third, intrinsic donor-to-donor variations make it difficult to determine the effects of deficient *SMS* expression. To overcome these limitations, we used MSCs derived from healthy male donors, which were genetically engineered to silence *SMS* gene expression. The advantages to this approach are that each experiment was carried out with cells derived from multiple different donors ensuring reproducibility, fresh bone marrow from healthy donors was readily available, and importantly, we were able to use the isogenic control to account for differences that arise from donor to donor variations (comparing side-by-side the same cells with or without deficient *SMS* expression).

Here we report the effects of silencing *SMS* in MSCs via shRNAs using lentiviral vectors. To elucidate molecular alterations, we compared the transcriptome and metabolome of the cells. Both approaches suggest dysregulation of glucose metabolism, which was further associated with mitochondrial defects.

## Results

### Silencing SMS reduces cell proliferation but does not affect apoptosis

**Figure 1:**
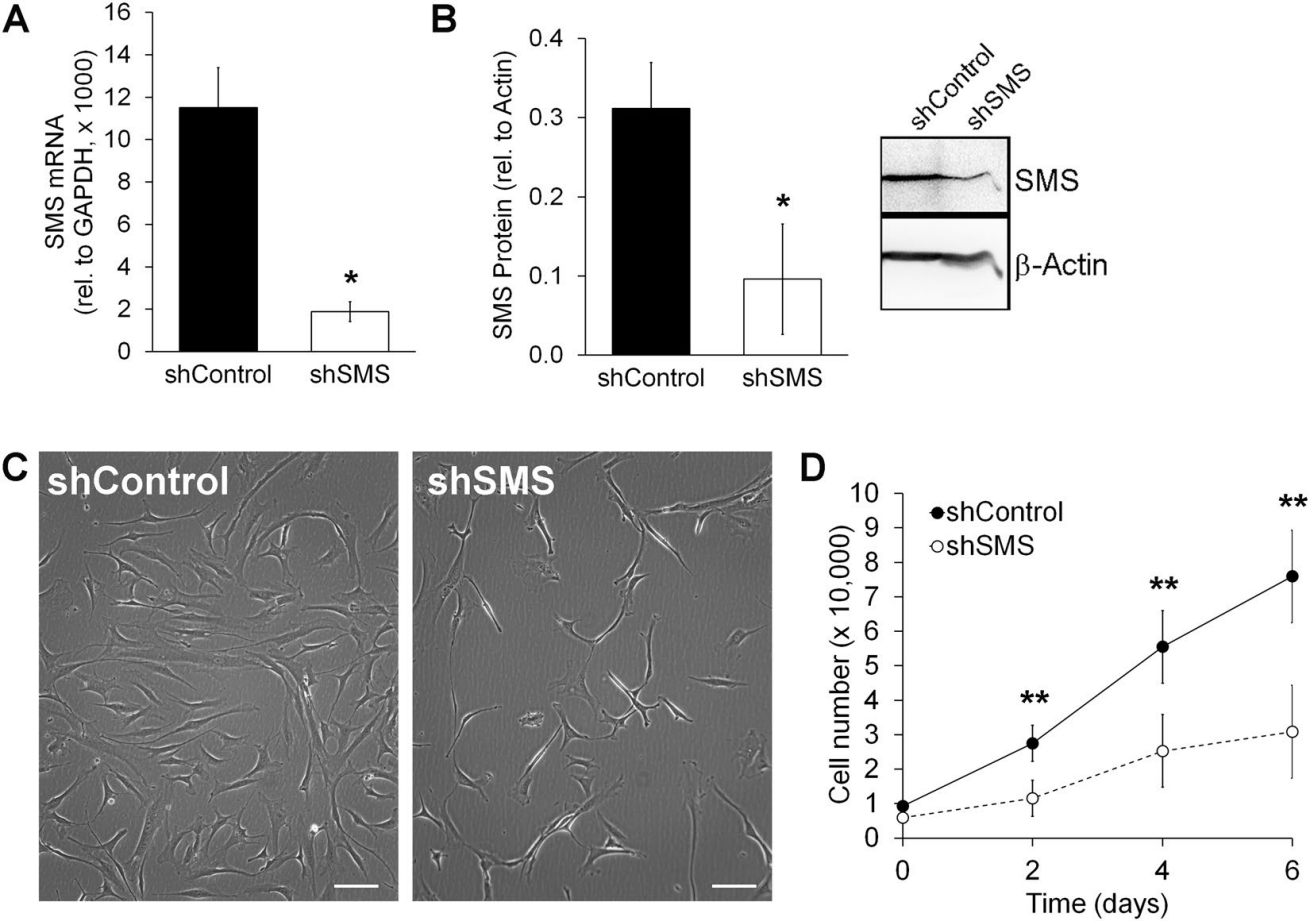
Silencing SMS causes morphological changes and inhibits proliferation in MSCs. (**A**) Real time PCR of MSCs transduced with either shControl or shSMS (n = 3). (**B**) Western Blot quantification also shows a decrease in SMS (band at 45 kDa) at protein levels (n = 3). (**C**) Representative phase-contrast images of MSCs transduce with either shControl or shSMS. Scale bar = 100 μm. (**D**) Proliferation curve with transduced cells (n = 7). Statistical differences were calculated using paired Student’s *t* test for each time point, where *p < 0.05 and **p < 0.005.

To cause SMS deficiency in MSCs, cells were transduced with lentiviral vectors constructed to either express an shRNA that blocks translation of *SMS* (shSMS), or an shRNA that does not bind to any human gene (shControl). As shown in Fig. 1A,B, transduction of MSCs with shSMS leads to an efficient reduction of SMS at the mRNA and protein levels, as compared to MSCs transduced with shControl.

Compared to controls, MSCs transduced with shSMS changed their morphology, becoming smaller and apparently less adherent, as suggested by the light refraction on the cell edges under a phase contrast microscope (Fig. 1C). Notably, cell proliferation was reduced by 2.3-fold (p < 0.005) upon silencing SMS (Fig. 1D and Figure S1A). We also tested if silencing SMS could increase cell death. However, no effect on apoptosis was evident: MSCs with shSMS could be cultured for at least 28 days (Figure S1B) and use of an apoptosis array kit showed no significant differences on 12 detected proteins, in between MSCs transduced with shControl and shSMS (Figure S1C). These results suggest that SMS is not required for cell survival, but strongly affects the proliferative potential of MSCs.

### Silencing SMS inhibits osteogenesis

**Figure 2:**
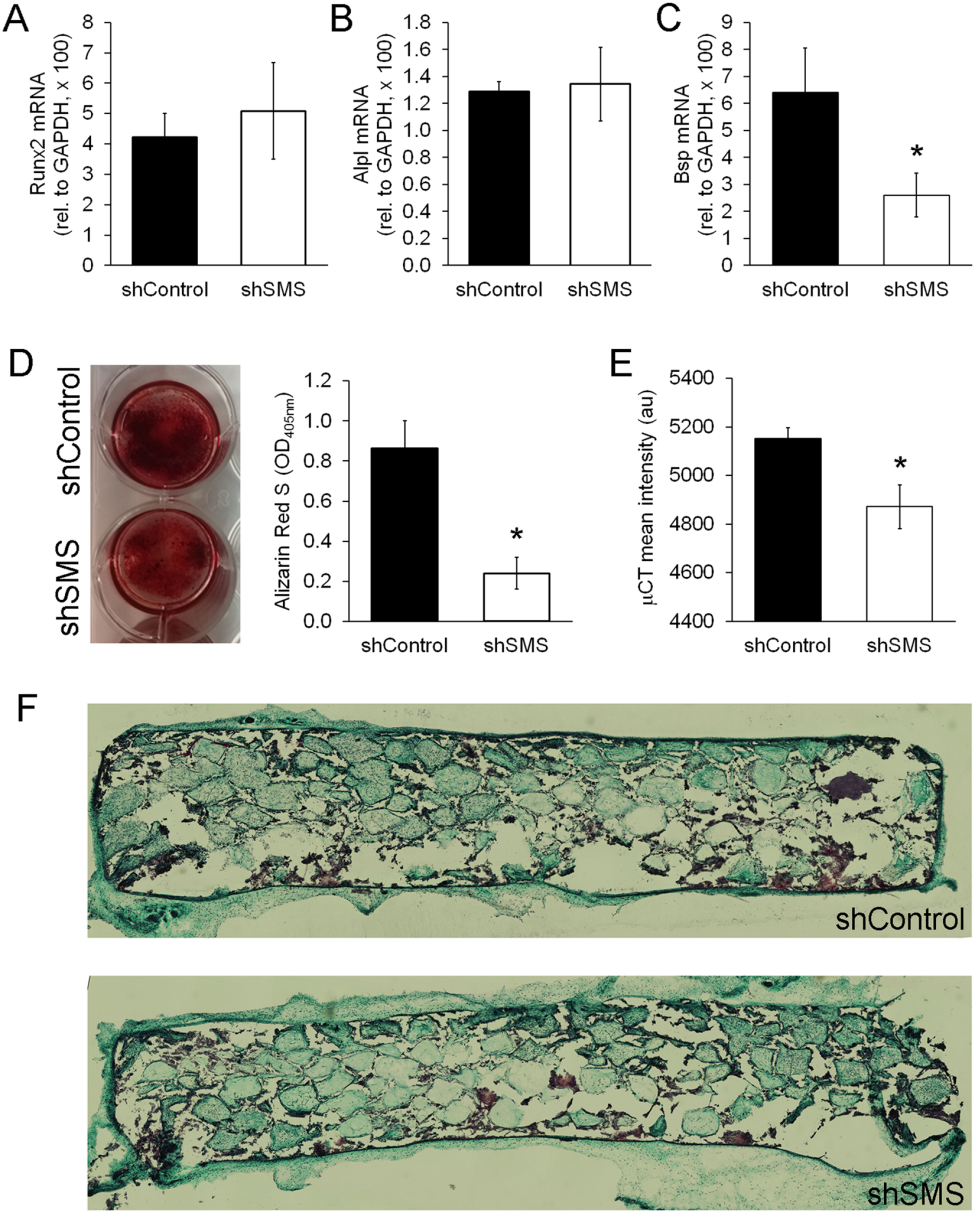
Silencing SMS inhibits osteogenesis of MSCs. (**A**) Runx2 mRNA levels measured after 1 day in osteogenic media, (n = 4). (**B**) Alpl mRNA measured at day 14 (n = 3). (**C**) Bsp mRNA, also measured at day 14 (n = 5). (**D**) Alizarin Red S staining measured after 28 days in osteogenic media (n = 4). Image shows representative wells after staining. (**E**) μCT measurements in MSC-containing HA/PLG scaffolds, 8 weeks after implantation in NSG mice (n = 7, with MSCs derived from 2 different donors). (**F**) Representative images of Masson’s trichrome staining on sagittal sections of scaffolds, 8 weeks after implantation in NSG mice. Cartilage is violet/dark blue, mineralized bone is blue/green, and unmineralized bone in red. Statistical differences were calculated using paired Student’s *t* test, where *p < 0.05.

To investigate if SMS deficiency could affect osteogenesis, we measured expression of osteogenic markers at different time points, according to the differentiation stages of the cells^6^. At day 1 (commitment), mRNA levels of transcription factors Runx2 and Sp7 (Osterix) were not altered by shSMS (Fig. 2A and not shown). At day 14 (maturation phase), no effect on alkaline phosphatase (ALP) levels were detected (Fig. 2B). However, bone sialoprotein (Bsp) was 2.5-fold downregulated by shSMS (p < 0.05) at this time point (Fig. 2C), suggesting that inefficient SMS expression affects maturation, rather than the commitment of MSCs becoming osteoblasts. This impaired maturation correlates with a strong 3.6-fold reduction in mineralization (p < 0.05; measured at day 28) in SMS-deficient MSCs, as compared to controls (Fig. 2D).

Next, we tested if silencing SMS would also affect osteogenic differentiation of MSCs *in vivo*. Here, MSCs (shControl or shSMS) were seeded into HA/PLG scaffolds^19^ and implanted bilaterally and subcutaneously into immune deficient NOD/SCID IL-2Rγ^−/−^ (NSG) mice. 8 weeks after, mice were humanely euthanized and ectopic bone formation was measured by micro-Computed Tomography (μCT). As shown in Fig. 2E, our results indicate that silencing SMS in MSCs leads to reduced mineral density *in vivo* (p < 0.05). However, histological analysis using Masson’s Trichrome staining showed no evident differences in between conditions, suggesting that in this model, silencing SMS in MSCs only mildly reduces bone formation. Noticeably, the lower levels of bone formation with shSMS was not due to the loss of cells, as images taken with an epi-fluorescence microscope show that cells expressing tdTomato (i.e. human MSCs) were equivalently present in scaffolds with MSCs with shControl and shSMS (Figure S2). Altogether, our results show that silencing SMS in MSCs causes a reduction in bone formation, both i*n vitro* and *in vivo*.

### Silencing SMS alters metabolite levels

To examine how SMS deficiency changes metabolites within MSCs, we transduced cells derived from 4 different donors with either shControl or shSMS and examined their metabolic content using gas chromatography - time of flight mass spectrometry (GCTOF MS). A total of 564 metabolites were detected in each sample. From these, 168 (30%) are annotated and therefore have a common name. The remaining 70% of metabolites are only referenced by a BinBase number^7^ and consequently their cellular functions are largely unknown. Among all detected metabolites, we found 33 molecules increased and 12 decreased in MSCs with shSMS as compared to controls (shControl) (Table 1 and Table S1). In line with findings reported from MSCs derived from SRS patients^5^, we found that shSMS leads to an accumulation of spermidine, while spermine was undetected in all samples, most likely because spermine was below detection levels. We also found a decrease of putrescine (the spermidine precursor), suggesting that MSCs respond to imbalanced polyamines by regulating compensatory mechanisms (Figure S3).

**Table 1:** Differentially expressed metabolites in MSCs transduced with shControl or shSMS.

Metabolite	shControl	shSMS	shControl/shSMS	Description/function
Phosalone	819	1983	0.4	?
210347	3621	7896	0.5	?
glucose	295124	626112	0.5	glucose metabolism
TG(i-14:0/22:0/a-15:0)	198	409	0.5	lipid
210694	301748	599831	0.5	?
135862	2813	5174	0.5	?
16747	689	1258	0.5	?
TG(12:0/19:0/i-20:0)	7396	13480	0.5	lipid
n-acetyl-d-hexosamine	780	1413	0.6	lysosomal
Trimethylpyrazine	1383	2500	0.6	?
Mepyramine	2511	4447	0.6	?
2-Methyl-1-phenyl-2-propanyl butyrate	3039	5256	0.6	?
4-Ethoxybenzaldehyde	4215	6958	0.6	?
spermidine	19065	30108	0.6	polyamine
cytidine	5160	7881	0.7	nucleoside
fructose	67756	98949	0.7	glucose metabolism
tagatose	99500	142710	0.7	glucose metabolism
TG*	3064	4344	0.7	lipid
TG(21:0/20:0/12:0)	8362	11786	0.7	lipid
carboxylic acid*	2104	2790	0.8	?
6-O-Oleuropeoylsucrose	1231	1625	0.8	saccharolipid
187855	3032	3991	0.8	?
170993	1003	1296	0.8	?
CL*	560	720	0.8	mitochondrial lipid
hexaric acid	442	555	0.8	?
5-Methylquinoxaline	4421	5530	0.8	?
CL**	4613	5763	0.8	mitochondrial lipid
171564	68866	84439	0.8	?
13-cis Retinol	16208	19807	0.8	retinoid
TG(18:1(9Z)/18:1(9Z)/18:1(9Z))	3737	4368	0.9	lipid
citric acid	211528	245437	0.9	glucose metabolism
phenylalanine	445510	509173	0.9	essential aminoacid
Cyclohexyl Acetate	3205	3548	0.9	?
Licoagrochalcone D	6231	5294	1.2	?
fumaric acid	119210	99758	1.2	glucose metabolism
malate	186445	155946	1.2	glucose metabolism
TG(16:0/20:2n6/16:1(9Z))	18226	15169	1.2	lipid
CL(8:0/14:0/a-17:0/23:0)	42758	34792	1.2	mitochondrial lipid
TG(a-17:0/12:0/i-20:0)[rac]	16109	13028	1.2	lipid
3 hydroxy-3-methylglutaric acid	1412	1085	1.3	leucine degradation
4-hydroxybutyric acid	1162	874	1.3	GABA precursor
2-hydroxyglutaric acid	7569	5596	1.4	glucose metabolism
alpha-aminoadipic acid	6519	4317	1.5	lysine pathway
CL(8:0/a-17:0/i-18:0/a-21:0)	31855	18930	1.7	mitochondrial lipid
putrescine	107636	52541	2.0	polyamine

A succinct description of their biological context is included when available. Metabolites identified by numbers (BinBase) do not have a common name. Abbreviations: TG = triglyceride, CL = cardiolipin. TG* = TG(15:0/22:6(4Z,7Z,10Z,13Z,16Z,19Z)/ 22:6(4Z,7Z,10Z,13Z,16Z,19Z)), carboxylic acid* = 3,4,5-trihydroxy-6-oxane-2-carboxylic acid, CL* = CL(16:0/20:4(5Z,8Z,11Z, 14Z)/18:2(9Z,11Z)/18:2(9Z,11Z)), CL** = CL(16:1(9Z)/18:2(9Z,11Z)/16:1(9Z)/18:2(9Z,12Z)). See complete metabolomic analysis in Table S1.

Interestingly, among the differentially expressed metabolites, many were associated with lipids, mitochondrial lipids, and glucose metabolism. For example, fructose, glucose and citric acid were increased in cells with shSMS, while malate and fumaric acid were reduced, as compared to controls, suggesting an impaired citric acid cycle. Therefore, the results from our metabolome analysis confirmed dysregulation of the polyamine pathway and suggested dysfunctional mitochondria in MSCs with deficient SMS expression.

### Silencing SMS alters the gene expression profile of MSCs

**Figure 3:**
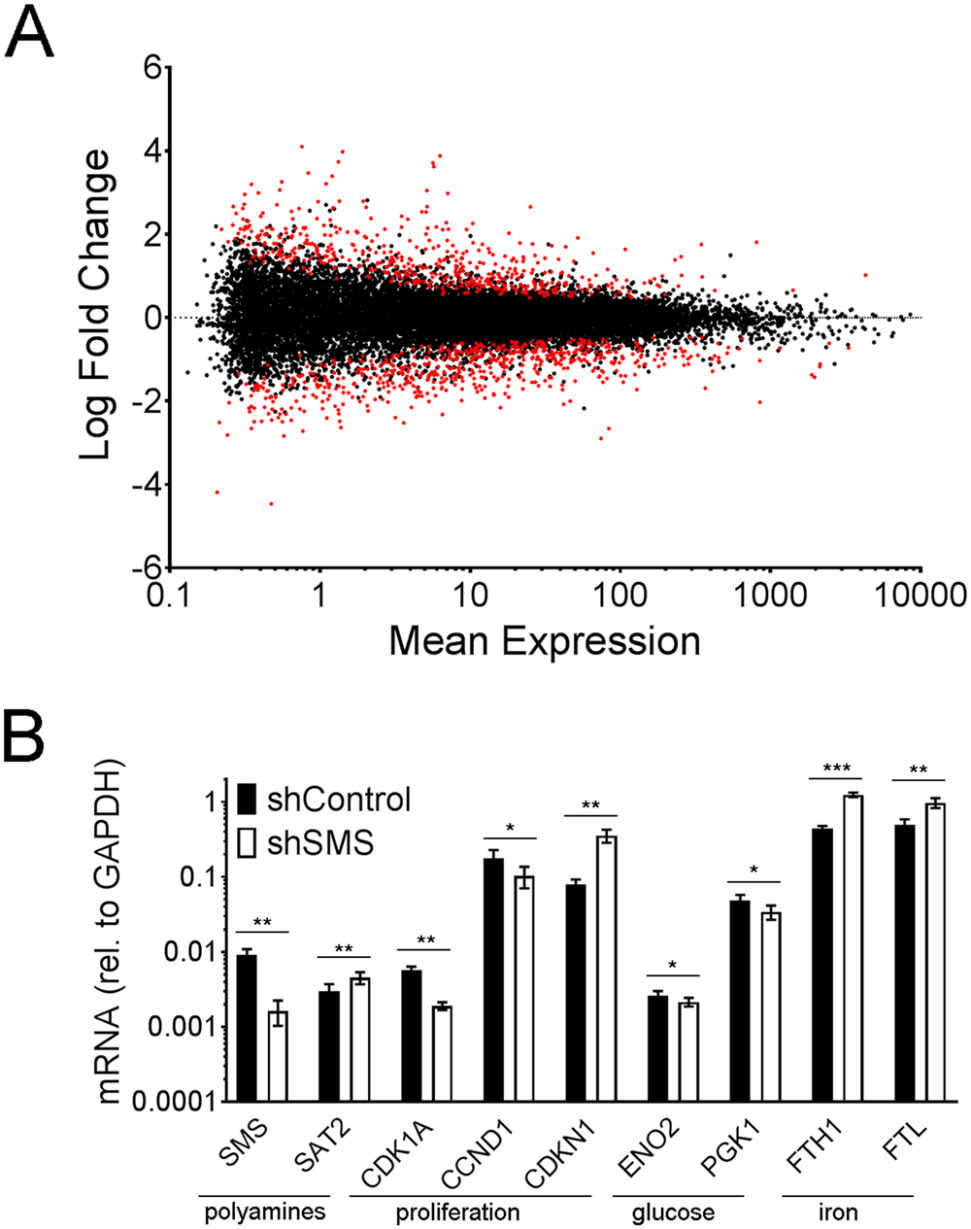
Silencing SMS strongly alters gene expression. (**A**) MA plot of differential gene expression, as measured using Tag-seq. Dots in red indicate gene expression levels that are statistically significant different. See Table S2 for complete list of genes (n = 4). (**B**) Confirmation by real time PCR of selected differentially expressed genes (n = 6). Statistical differences were calculated using paired Student’s *t* test, where *p < 0.05, **p < 0.005 and ***p < 0.0005.

To examine how SMS deficiency alters the transcriptome of cells, we compared the gene expression profile of MSCs derived from four different donors, transduced with either shControl or shSMS (Fig. 3A and Table S2). Tag-seq analysis showed 1084 genes significantly (p < 0.05) dysregulated between conditions. Many of these genes are related to cell cycle progression. However, we also found differential expression of genes involved with polyamines and glucose metabolism. Selected differentially expressed genes were confirmed by real time PCR (Fig. 3B). The observed downregulation of SMS is rather confirmatory, but the increase of Spermidine/Spermine N1-Acetyltransferase Family Member 2 (SAT2) was unexpected and may represent a compensatory mechanism to reduce Spermidine levels (which are increased, as shown in Table 1). An increase of Cyclin Dependent Kinase Inhibitor 1 A (CDKN1) and a decrease of both Cyclin Dependent Kinase 1 (CDK1A) and Cyclin D1 (CCND1) are very much in line with the observed inhibition of cell proliferation. Downregulation of Enolase 2 (ENO2) and Phosphoglycerate Kinase 1 (PGK1) correlate with impaired glucose metabolism.

Finally, we confirmed a strong increase in gene expression of both ferritin heavy chain (FTH) and ferritin light chain (FTL), suggesting that deficient SMS expression is causing an imbalance of iron metabolism. In summary, silencing SMS causes strong changes in gene expression, which are in line with the described alterations in cell proliferation, polyamines and glucose metabolism.

### Silencing SMS causes mitochondrial dysfunction

It has been suggested that impaired SMS expression leads to increased oxidative stress^3^. To test if this is also the case in human MSCs, we stained transduced cells with DCFDA, which is an indicator of reactive oxygen species (ROS). However, 93% of transduced cells (i.e. expressing tdTomato) did not stain for DCFDA (conjugated to FITC), while 83% of DCFDA positive cells did not express tdTomato. This result suggests that silencing SMS may even cause a reduction of ROS in MSCs (Figure S4).

**Figure 4:**
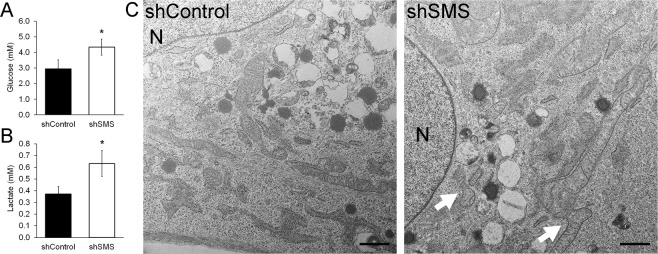
Silencing SMS affects mitochondrial function. Transduced MSCs were cultured in serum-free media containing 5.5 mM glucose and no lactose. After 24 hours, supernatants were collected to determine levels of glucose (**A**) and lactose (**B**) (n = 7). Statistical differences were calculated using paired Student’s *t* test, where * p < 0.05. (**C**) Transduced MSCs as seen under TEM. At least 10 cells per condition were examined in detail, finding consistently more fusing mitochondrion (arrows) in SMS-deficient cells (shSMS). N = nucleus. Scale bar = 1 μm.

The above mentioned alterations in glucose metabolism, as suggested by metabolome and transcriptome analysis, imply impaired mitochondrial function. This notion was further confirmed as silencing SMS reduced glucose consumption, but increased lactate secretion (the product of glycolysis when the citric acid cycle is impaired) (Fig. 4A,B). Additionally, inspection of MSCs at an ultrastructural level using transmission electron microscopy (TEM) showed an apparent higher number of cells with mitochondrial fusion in MSCs transduced with shSMS (7 out of 10 cells) as compared to MSCs with shControl (2 out of 11 cells, Fig. 4C). These results suggest that silencing SMS leads to severe mitochondrial dysfunction and we hypothesize that this is the underlying cause for inhibited proliferation and osteogenic differentiation in MSCs. In line with this idea, we and others have shown that incubation under hypoxia (i.e. inhibiting mitochondrial function) leads to both reduced proliferation and inhibited osteogenesis^8^.

**Figure 5:**
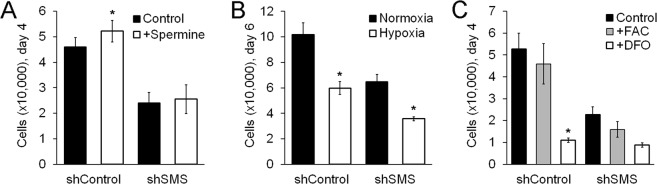
Supplementation with spermine, hypoxia, FAC, or DFO does not rescue the inhibited proliferation of MSCs caused by silencing SMS. Proliferation assays of transduced MSCs. In all experiments, MSCs with shSMS were significantly less, as compared to MSC with shControl (as shown in Fig. 1). (**A**) MSCs cultured for 4 days in the presence of 1 mM aminoguanidine (to inhibit amine oxidase contained in FBS), and with or without spermine (5 μM) (n = 6). (**B**) MSCs cultured for 6 days in either normoxia (20.5% Oxygen) or hypoxia (1% Oxygen) (n = 3). (**C**) MSCs cultured for 4 days with either no supplements (Control), FAC (100 μg/ml), or DFO (100 μM), (n = 3). Statistical differences were calculated using paired Student’s *t* test, where *p < 0.05.

Finally, we examined three different approaches to restore cell proliferation in SMS-deficient MSCs. First, we tested supplementation with spermine, the catalytic product of SMS. Addition of spermine (5 μM) caused a mild increase in cell proliferation, which was not significant in MSCs transduced with shSMS (Fig. 5A). Second, we tested hypoxic pre-conditioning (to minimize mitochondrial function). Here, culture under hypoxia (1% Oxygen), led to reduced proliferation of MSCs (shControl), to levels comparable to the effect of silencing SMS. However, MSCs with shSMS under hypoxia showed a further inhibition of proliferation (Fig. 5B). Finally, since MSCs with shSMS showed increased expression of ferritin (both, heavy chain and light chain subunits, Fig. 3B), and ferritin acts by storing iron, we examined the effect of supplementing cells with either iron accessible to cells as ferric ammonium citrate (FAC) or an iron chelate (deferoxamine, DFO). As shown in Fig. 5C, DFO (100 μM), similarly to hypoxia, caused a strong inhibition of cell proliferation, while supplementation with FAC (100 μg/ml) had no evident effect. These results suggest that supplementation with spermine is insufficient to restore proliferation of SMS-deficient MSCs, mitochondrial dysfunction is unlikely the only cause for reduced proliferation in SMS-deficient MSCs, and modulation of iron levels is insufficient to restore the effect of SMS deficiency in MSCs. Therefore, finding alternative strategies to rescue MSCs with impaired SMS expression remains a key challenge, to develop a treatment for the osteogenic defects of SRS patients.

## Discussion

Atraumatic osteoporotic fractures commonly occur in SRS patients, impairing quality of life^5^. It is therefore critical to elucidate the causes for how deficient SMS expression impairs bone formation. Our results establish a correlation between deficient SMS expression and several molecular changes that could account for impaired osteogenesis by bone marrow-derived MSCs. The notion of modeling the disease by using MSCs derived from healthy donors and transducing them with a specific shRNA allowed the precise dissection of the role of SMS, while minimizing variations that are intrinsic from donor-to-donor. In addition, it permitted inclusion of more replicates than what would have been feasible to collect from the rare SRS patients. Nonetheless, confirmation of our results in MSCs derived from SRS patients remains an important task.

The observed effect of SMS deficiency on osteogenesis is in line with the described lower mineralization observed in MSCs derived from SRS patients^5^. In addition, our work suggests that the inhibition occurs during osteoblasts’ maturation phase^6^ because bone sialoprotein (BSP) was markedly reduced, even before any mineralization became evident. Importantly, mineralization was also reduced *in vivo*, suggesting that SMS deficiency leads to reduced bone formation, even under physiologically relevant osteogenic stimuli. We hypothesize that in our employed mouse model the effect of SMS deficiency seems mild because much of the scaffold becomes populated by endogenous (murine) cells (see DAPI staining in Figure S2), suggesting that at least partially the ectopic bone formation is driven by cells other than the transplanted human MSCs. Since our work suggests that lower bone volume of SRS patients may be attributed to inhibited proliferation of osteoprogenitor cells (MSCs), future studies should also consider a sub-population of MSCs prior to any cell expansion, recently described as skeletal stem cells^9^.

Due to the lack of SMS activity, SRS patients show a characteristic increase of spermidine and decrease of spermine^1^. Our metabolome analysis confirmed the increase of spermidine, but spermine, although measured, was below detection levels. This result raises two concepts: First, the negative effects of SMS-deficiency could be due to accumulation of spermidine, rather than due to lack of spermine (which is already very low). Second, spermine might be very limited in MSCs, making these cells especially susceptible to decreased spermine levels.

Supporting the notion of toxicity due to high spermidine, MSCs with shSMS showed a decrease of putrescine, which is a main spermidine precursor^10^, implying a potential compensatory mechanism. This variation in both putrescine and spermidine was also recently described in lymphoblastic cell lines derived from SRS patients, as compared to healthy donors^11^. Also ornithine (a main putrescine precursor) was consistently decreased in all four investigated MSCs (Table S1 and Figure S3), although the magnitude was variable, leading to a lack of statistically significant differences. Remarkably, in MSCs the relative abundance of each polyamine correlates with their synthesis order, with ornithine being the most abundant, and spermine the least abundant (Figure S3).

SMS deficiency led also to a significant increase of SAT2 mRNA. This gene is a paralog of SAT1, and codes for an enzyme that catalyzes the conversion of spermine or spermidine into acetylspermine or acetylspermidine, respectively. The increase of SAT2 is therefore likely an additional compensatory mechanism to reduce spermidine levels. Finally, it should be noted that supplementation with spermine promotes osteogenesis in MSCs, supporting the notion that this polyamine, although low in MSCs, might be essential for proper osteoblastic differentiation^12,13^. However, in our experimental setting, supplementation with spermine only minimally increased proliferation of MSCs, which could be due to limited internalization of spermine into the cells.

Li *et al*. showed that in fruit flies SMS deficiency causes oxidative stress^3^, and oxidative stress reduces both proliferation and osteogenesis of MSCs^14,15^. However, we did not find evidence for increased oxidative stress due to SMS deficiency. In fact, transduced cells with shSMS (i.e. expressing tdTomato) tend to show lower DCFDA staining, suggesting a decrease of reactive oxygen species in SMS-deficient cells. In line with this finding, Tag-seq analysis showed a significant increase of Glutathione Peroxidase 1 (Gpx1), which catalyzes the reduction of ROS (Table S2).

The differentially expressed metabolites and transcripts were indicative of deficient mitochondrial function, which was confirmed by decreased glucose consumption, increased lactate secretion, and high mitochondrial fusion (Fig. 4). Of note, mitochondrion are major users of cellular iron, play a major role in iron metabolism, and rely on iron transport, storage, and regulatory proteins to maintain iron homeostasis^16^. In this context, the increase of ferritin mRNAs (both heavy chain and light chain) is likely in response to an imbalance of iron. In fact, it was recently proposed that iron and polyamines’ metabolism are intimately linked^17^. However, reducing mitochondrial function by exposing the cells to hypoxia led to a further reduction in cell proliferation, suggesting that mitochondrial function alone is not the only variable responsible for inhibited cell growth. Similarly, the iron chelate DFO had a very similar effect to hypoxia on MSCs, i.e. further repressing cell proliferation. In fact, DFO is commonly used to mimic hypoxia, as it leads to stabilization of the transcription factor hypoxia-inducible factor 1 (HIF-1).

Altogether, this work offers molecular insight into the alterations caused by SMS-deficiency in MSCs. These results may therefore contribute to the future development of potential treatments to improve bone formation in SRS patients.

## Methods

### MSC isolation and expansion

MSCs were isolated from bone marrow aspirates from healthy male donors, based on density and adherence to plastic, as previously described^18^. In brief, fresh bone marrow aspirates from healthy donors (StemExpress, cat# BMEDT050F) were mixed 1:1 with PBS, layered over Ficoll-Paque PLUS (GE Healthcare, cat# 17144003) and centrifuged for 30 minutes at 600 × *g*. Cells from the buffy coat were then plated into tissue culture flasks. Cells were cultured using MEM-α (HyClone, cat# SH3026501) supplemented with 10% fetal bovine serum (FBS; Atlanta Biologicals, cat# S11550). After 2 days, medium was changed to wash off non-adherent cells. Remaining cells were then expanded, acquiring the morphology, immune phenotype, and trilineage differentiation potential characteristic of bone marrow-derived MSCs^8^. During expansion, media was changed every 2–3 days. For all experiments, MSCs were used in between passages 3 and 6, where one passage is approximately equivalent to 3 population doublings.

### Generation of lentiviral vectors and transduction

To silence SMS, we used third generation lentiviral constructs with the general form: pCCLc-U6-shRNA-PGK-tdTomato-WPRE, where shRNA is either the sequence to an shRNA that does not target any human gene (shControl), or an shRNA to silence SMS (shSMS). The mRNA-targeting portion (RNAi) of shSMS is 5′- CTACCAGTGATACCATCTCTA-3′. Lentiviral vectors were packaged in Lenti-X 293 T cells by transfecting the cells with the plasmids of interest and packaging plasmids VSVG and Δ8.91, using TransIT-293 Transfection Reagent (Mirus Bio, cat# MIR 2705). Lentiviral transductions were performed using protamine sulfate (20 μg/ml) and with sufficient lentivirus to generate approximately 80% tdTomato positive cells, as determined using a microscope with epi-fluorescence, 3 days after transduction.

### Cell proliferation assays

For cell counting experiments, transduced MSCs were plated in triplicate for each time point and condition in 12-well plates (10,000 cells per well). The day after (day 0) and at day 3, media was changed to fresh MEM-α + 10% FBS, with or without supplements. Spermine (cat# 55513-100MG), Ammonium iron (III) citrate (FAC; cat# F5879-100G) and deferoxamine mesylate salt (DFO; cat# D9533-1G) were purchased from Sigma-Aldrich. At the indicated time points, cells to count were lifted using Trypsin and counted using Trypan Blue exclusion dye and hemocytometer.

As an orthologous method to measure cell proliferation we used a CyQUANT™ Direct Cell Proliferation Assay (Thermo Fisher, cat# C35011). Here, transduced MSCs were plated in triplicate into 96-well plates (500 cells per well). At each indicated time point, a plate was frozen at −80 °C. After the last time point, plates were treated with lysis buffer and a DNA-binding fluorescent dye as described by the manufacturer. Plates were measured by fluorescence at 520 nm emission.

### RNA isolation, reverse transcription, and real time PCR

RNA was isolated using the Direct-zol RNA Mini-Prep kit (Zymo Research, cat# R2051), following manufacturer’s instructions. For cDNA synthesis, the Taqman Reverse Transcription kit (Thermo Fisher, cat# N8080234) was used as described by the manufacturer. Real time PCR was performed using Taqman gene expression assays (Thermo Fisher) and Taqman Universal Master Mix reagents (Thermo Fisher, cat# A30865). The primers/probe are identified by the following numbers: GAPDH: Hs02758991_g1, SMS: Hs01924834_u1, RUNX2: Hs01047973, BSP: Hs00173720_m1, CDKN1: Hs00355782_m1, CCND1: Hs00765553_m1, PGK1: Hs00943178_g1, ENO2: Hs00157360_m1, SAT2: Hs01070426_g1, FTH1: Hs01000476_g1, and FTL: Hs00830226_gH.

### Western blot

Approximately 1 million transduced MSCs per condition were lifted using Trypsin treatment. Cells were then lysed in ice-cold RIPA buffer (Thermo Fisher, cat# 89900) containing a protease and phosphatase inhibitor cocktail (Thermo Fisher, cat# 78440). Cell lysates were mixed with Laemmli buffer (Bio-Rad, cat# 1610747) containing β-mercaptoethanol, and boiled at 95 °C for 5 minutes and subjected to SDS–PAGE. Proteins were transferred onto PVDF membranes and incubated overnight with primary antibodies against SMS (1:500, clone 1G6, Abnova, cat# H00006611-M01) and β-actin (1:2,000, Santa Cruz, cat# sc-47778). Secondary antibodies (1:2,000) conjugated to horseradish peroxidase (Santa Cruz, cat# sc-2005) were then added for 1 hour at room temperature, and proteins were detected and photographed using SuperSignal West Pico Chemiluminescent Substrate (Thermo Fisher, cat# 34580) and Image Lab software (Bio-Rad). Semi-quantification of protein levels was calculated by dividing pixel intensity of SMS by β-actin, measured using Adobe Photoshop CS6 software.

### Apoptosis array

To detect potential changes in apoptosis, a Proteome Profiler Human Apoptosis Array Kit was used following manufacturer’s instructions (R&D Systems, cat# ARY009). In brief, four days after transduction of MSCs with the respective lentiviral vectors, cells were lifted using Trypsin, washed once with PBS and lysed for protein extraction using the kit’s reagents and protocol. To semi-quantify protein levels, dots on developed membranes were analyzed for pixel intensity using Adobe Photoshop.

### Osteogenic differentiation *in vitro*

Transduced MSCs were seeded into 12-well plates at a density of 50,000 cells/well. The next day, medium was changed to osteogenic media (MEM-α + 10% FBS supplemented with 0.2 mM ascorbic acid, 0.1 μM dexamethasone, and 10 mM β-glycerolphosphate) and cultured for up to 28 days with medium changes every 3–4 days. RUNX2 and BSP mRNA levels were measured at days 1 and 14 respectively. At day 28, mineralization was measured using Alizarin Red S staining. In brief, cells were fixed with 10% v/v formalin solution for 15 minutes, washed once with PBS, stained with 1% w/v Alizarin Red S (ARS) indicator (Ricca Chemicals, cat# R0500000) for 20 minutes, washed twice with PBS and documented photographically. Then, wells were incubated with 10% v/v acetic acid for 30 minutes, scraped for further dissociation of cells, vortexed for 30 seconds and centrifuged at 12,000 × *g* for 15 minutes. Optic density of the supernatants was measured using a plate reader at 405 nm.

### Ectopic bone formation assay

All animal work was performed following approved protocols by the Institutional Animal Care and Use Committee (IACUC) at University of California, Davis. Transduced MSCs (shControl and shSMS) were seeded in Hydroxyapatite/Poly(Lactide-Co-Glycolide) (HA/PLG) scaffolds^19^, at 500,000 cells per scaffold (1 cm^2^). The next day, cell-containing scaffolds were implanted subcutaneously and bilaterally into immune deficient NOD/SCID IL-2Rγ^−/−^ (NSG) mice. Eight weeks after implantation, mice were humanely euthanized and ectopic bone formation was measured by micro-Computed Tomography (μCT) using an Inveon MM CT scanner (Siemens). Scans were then analyzed using Amide 1.0 software.

### Transcriptome analysis

After RNA extraction as described above, RNA samples were submitted to the Gene Expression Core at University of California, Davis. RNA quality was determined using Agilent Bioanalyzer 2100 (Agilent Technologies). cDNA library was built using 3′Tag-RNA-Seq library kit (Illumina, cat# 20020189). Sequencing was performed in one lane of a HiSeq 4000 platform with single-end 80 bp reads (SE80). Analysis (including quality control, mapping, multi-dimensional scaling (MDS) plot, and generation of tables of differential gene expression (DGE) was completed by the Bioinformatics Core at University of California, Davis. From these tables, an MA plot of DGE was generated using GraphPad Prism 7 software.

### Metabolome analysis

Metabolic profiling of MSCs with shControl and shSMS was performed as previously described^8^. In brief, 1 million cells per condition were lifted using Trypsin and submitted to the West Coast Metabolomics Center at University of California, Davis. There, samples were processed and analyzed by gas chromatography-time of flight mass spectrometry (GCTOF MS) as described^20^. Significant differences in levels of metabolites were identified using Excel, using a paired Student’s *t* test.

### Oxidative stress staining

Transduced MSCs with shControl or shSMS were incubated for 30 minutes with 5 μM cell permeable 2′,7′-dichlorodihydrofluorescein diacetate (DCFDA), (Fisher Scientific, cat# D399), washed three times with PBS, fixed for 15 minutes with formalin and directly inspected under a fluorescent microscope.

### Glucose and Lactate measurements

Transduced MSCs were cultured for 24 hours in serum free media. Then, supernatants were collected, stored at −80 °C and measured as described^8^. In brief, glucose and lactate concentrations of supernatants were determined using a Glucose Colorimetric/ Fluorometric Assay Kit (BioVision, cat# K606) and a Lactate Colorimetric Assay Kit (BioVision, cat# K607), respectively, following the manufacturer’s provided protocols.

### Transmission electron microscopy

Transduced cells were fixed using Karnovsky’s buffer and submitted to the Electron Microscopy Core at University of California, Davis. Here, samples were fixed again, dehydrated and embedded in resin. Sections were then cut using an ultra-microtome and mounted on a grid. Finally, sections were treated with heavy metals and inspected under a transmission electron microscope (Phillips). Over 10 cells per condition, derived from 2 different donors were inspected and documented for structural evidence of mitochondrial fusion.

### Ethical approval and informed consent

All experiments were performed strictly adhering to UC Davis policies, as stated in our approved Biological Use Authorization (BUA). Animal work was conducted in accordance with the protocol approved by UC Davis’ Institutional Animal Care and Use Committee (IACUC).

## Supplementary information

Supplementary Figures S1-S4

Table S1

Table S2

## Data Availability

All data associated to this manuscript is freely available as either main figures, supplementary files, or as deposited in publicly available databases.
