# Author Correction: Modeling Snyder-Robinson Syndrome in multipotent stromal cells reveals impaired mitochondrial function as a potential cause for deficient osteogenesis

**DOI:** 10.1038/s41598-019-54994-2

**Published:** 2019-12-06

**Authors:** Ashley L. Ramsay, Vivian Alonso-Garcia, Cutter Chaboya, Brian Radut, Bryan Le, Jose Florez, Cameron Schumacher, Fernando A. Fierro

**Affiliations:** 1 Institute for Regenerative Cures, University of California Davis, 2921 Stockton Blvd, Sacramento, CA USA; 2 Department of Cell Biology and Human Anatomy, University of California, Davis, USA

Correction to: *Scientific Reports* 10.1038/s41598-019-51868-5, published online 28 October 2019

The original version of this Article contained a typographical error in the Abstract.

“Patients with Snyder-Robinson Syndrome (SRS) exhibit deficient Spermidine Synthase (SMS) gene expression”

now reads:

“Patients with Snyder-Robinson Syndrome (SRS) exhibit deficient Spermine Synthase (SMS) gene expression”

This has now been corrected in the PDF and HTML versions of the Article.

