# Supplementary material for: Modeling Snyder-Robinson Syndrome in multipotent stromal cells reveals impaired mitochondrial function as a potential cause for deficient osteogenesis
Source: Sci Rep. 2019 Oct 28;9:15395. doi: 10.1038/s41598-019-51868-5 (PMC6817887; doi:10.1038/s41598-019-51868-5)
Supplement: Supplementary file 1 — Supplementary Figures S1-S4 [file 41598_2019_51868_MOESM1_ESM.docx]

**Modeling Snyder-Robinson Syndrome in multipotent stromal cells reveals impaired mitochondrial function as a potential cause for deficient osteogenesis**

Ashley L. Ramsay, Vivian Alonso-Garcia, Cutter Chaboya, Brian Radut, Bryan Le, Jose Florez, Cameron Schumacher, Fernando A. Fierro.

**Supplemental Information**

**
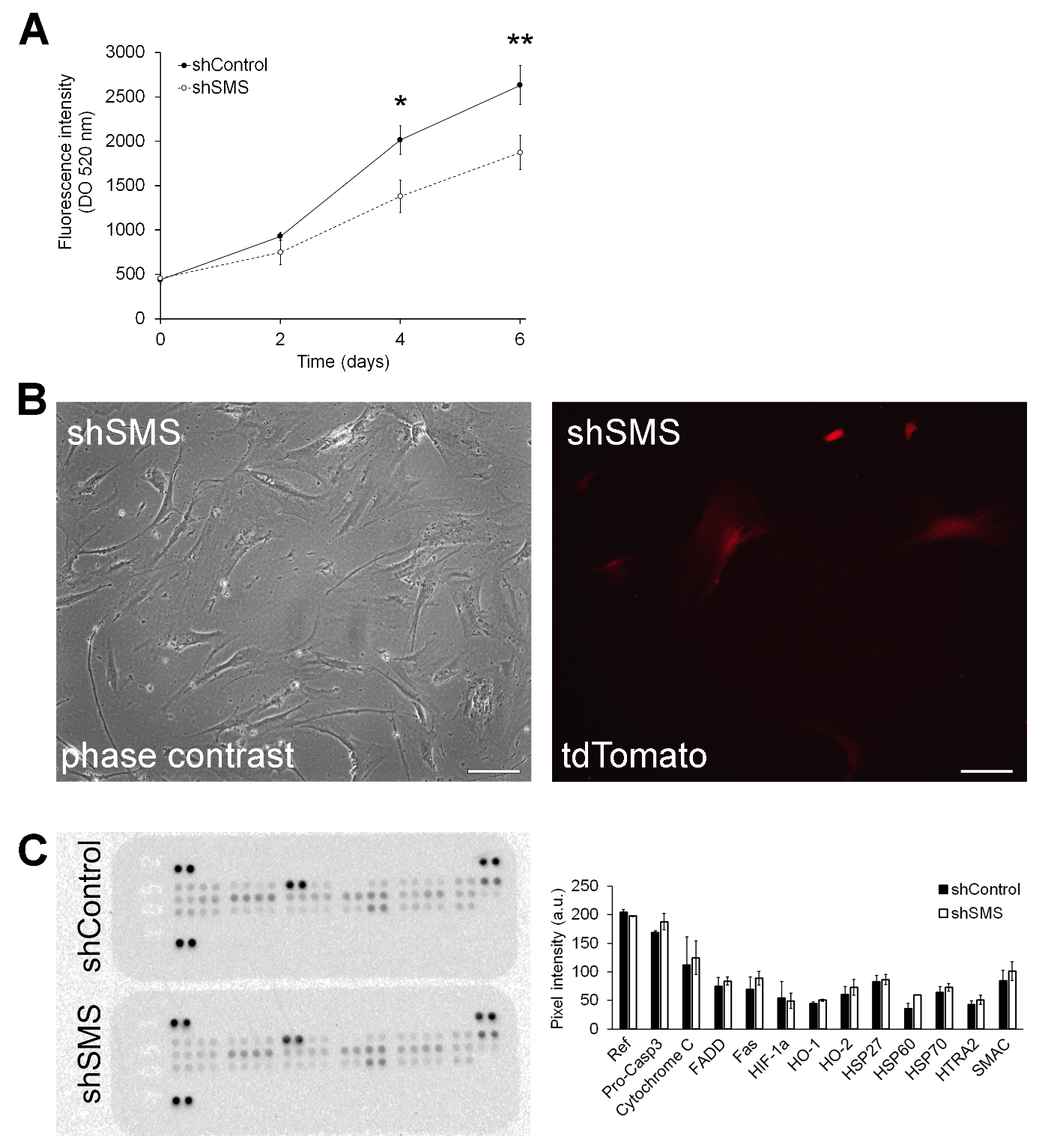
Figure S1. Silencing SMS does not impact cell viability.** (A) Cell proliferation assay (CyQUANT) confirming reduced proliferation of MSCs with shSMS, as compared to MSCs with shControl (n = 5). (B) Representative phase contrast and fluorescent images of MSCs transduced with shSMS, 28 days after transduction. Although non-transduced cells predominate, transduced cells (i.e. tdTomato positive cells) are still present, suggesting that silencing SMS is not directly causing cell death. (B) Representative membranes from a Human Apoptosis Array Kit (cat# ARY009) incubated with cell lysates from MSCs transduced with either shControl or shSMS. For detailed description of each dot, please refer to the manufacturer’s product datasheet. Histogram on left shows average pixel intensity of detected proteins (n = 4). Statistical differences were calculated using paired Student’s *t* test, where * p <0.05 and ** p < 0.005.

**
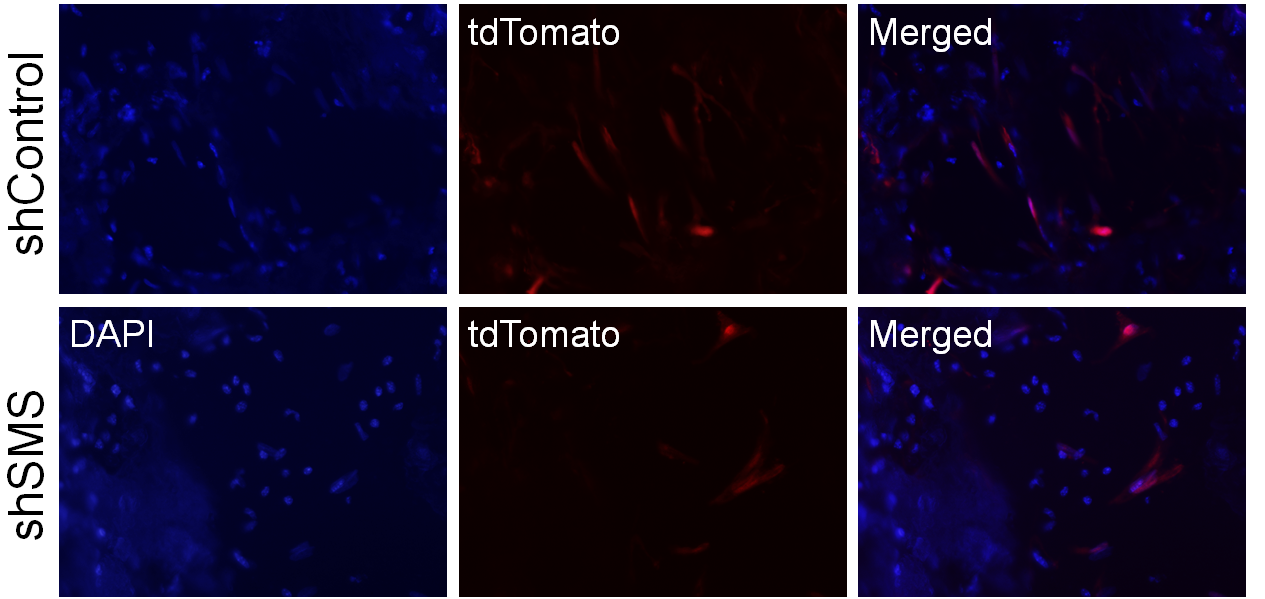
Figure S2. MSCs remain present in scaffolds, 8 weeks after implantation.** Immunofluorescent staining on sections from scaffolds, 8 weeks after implantation into NSG mice. Total nuclei are stained with DAPI (blue) and human cells are detected by expression of tdTomato (red).

**
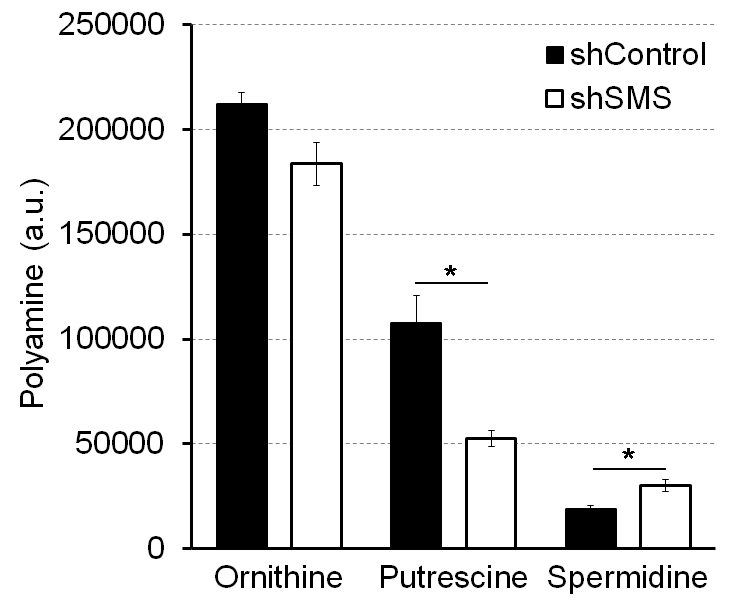
**

**Figure S3. Polyamine levels detected by GCTOF MS.** Levels of polyamines measured by mass spectrometry (relates to Table 1 and Table S1). Notice that in the polyamine pathway, ornithine is the precursor of putrescine, putrescine is the precursor of spermidine, and spermidine is the precursor of spermine (n = 4). Spermine is not shown, as it was below detection levels. Statistical differences were calculated using paired Student’s *t* test, where * p <0.05.

**
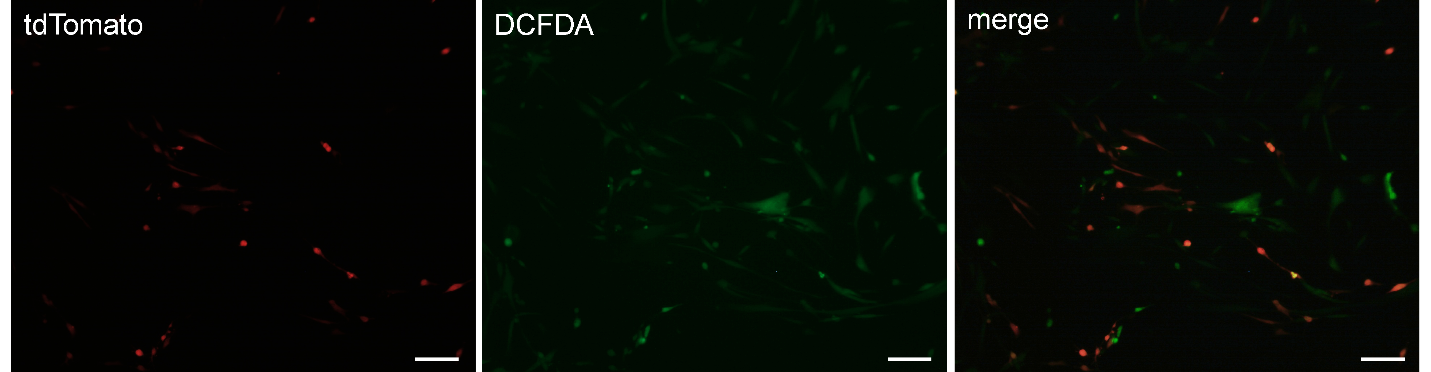
Figure S4. Silencing SMS does not increase reactive oxygen species in MSCs.** MSCs transduced with shSMS (containing reporter tdTomato - red) were incubated with DCFDA (green). Merged image suggests that in most cells, highest levels of ROS (green) are not in transduced cells (red).
